# 

**Published:** 1886-07

**Authors:** 


### COLLABORATORS.

CHICAGO:

PROFESSOR J. A. ALLEN, M. D., LL. D.

PROFESSOR E. ANDREWS, M. D., LL. D.

PROFESSOR JOHN BARTLETT, M. D.

PROFESSOR W. H. BYFORD, A. M., M. D.

PROFESSOR O. C. DEWOLF, A. M., M. D.

PROFESSOR E. C. DUDLEY, A. B., M. D.

PROFESSOR J. H. ETHERIDGE, A. M., M. D

PROFESSOR C. FENGER, M. D.

PROFESSOR J. N. HYDE, A. M., M. D.

PROFESSOR E. F. INGALS, A. M., M.D.

PROFESSOR W. W. JAGGARD, A. M., M. D.

PROFESSOR H. A. JOHNSON, M. D., LL. D.

PROFESSOR CHARLES GILMAN SMITH, A. M., M.D.

MILWAUKEE, WISCONSIN:

PROFESSOR N. SENN, M. D.

WASHINGTON, D. C.:

S. O. RICHEY, M. D.

UNITED STATES ARMY:

SURGEON, D. L. HUNTINGTON, M. D.

UNITED STATES NAVY:

MEDICAL DIRECTOR, J. M. BROWNE, M. D.

MEDICAL DIRECTOR, A. L. GIHON, A. M., M. D.

---

### CONTRIBUTORS OF ORIGINAL ARTICLES.

H. D. SCHMIDT,
A. K. VAN HORN,
W. H. FORWOOD,
F. E. WAXHAM,
L. L. MAC ARTHUR
SCOTT HELM,
E. C. MANN,
JOHN BARTLETT,
W. E. CASSELBERRY,
H. J. REYNOLDS,
A. SCHIRMER,
HAROLD N. MOYER,
C. W. EARLE,
A. V. PARK,
F. BILLINGS,
A. J. OCHSNER,
O. C. DEWOLF,
A. E. HOADLEY,
F. C. DARNALL,
J. ZEISLER,
S. O. RICHEY.

SUBSCRIPTION PRICE, $3.00 PER YEAR.

Number, 318. JULY, 1886. Vol. LIII., Number 1.

LIBRARY
INDEXED
S.G.O.

THE

### [illegible]ICAGO MEDICAL JOURNAL AND EXAMINER

EDITORS:

S. J. JONES, M.D., LL.D.

W. JAGGARD, A.M., M.D. HAROLD N. MOYER, M.D.

COLLABORATORS.

CHICAGO:

PROFESSOR J. A. ALLEN, M. D., LL. D.
PROFESSOR E. ANDREWS, M. D., LL. D.
PROFESSOR W. H. BYFORD, A. M., M. D.
PROFESSOR O. C. DEWOLF, A. M., M. D.
PROFESSOR E. C. DUDLEY, A. B., M. D.
PROFESSOR C. FENGER, M. D.
PROFESSOR J. N. HYDE, A. M., M. D.
PROFESSOR E. F. INGALS, A. M., M.D.
PROFESSOR H. A. JOHNSON, M. D., LL. D.
CHARLES GILMAN SMITH, A. M., M.D.
PROFESSOR J. H. ETHERIDGE, A. M., M. D.

MILWAUKEE, WISCONSIN:
PROFESSOR N. SENN, M. D.

WASHINGTON, D. C.:
S. O. RICHEY, M. D.

UNITED STATES ARMY:
SURGEON, D. L. HUNTINGTON, M. D.

UNITED STATES NAVY:
MEDICAL DIRECTOR, J. M. BROWNE, M. D.
MEDICAL DIRECTOR, A. L. GIHON, A. M., M. D.

